# Diagnostic considerations in the clinical management of sudden swelling of the knee: a case report and review of the literature

**DOI:** 10.1186/s13256-023-04336-8

**Published:** 2024-01-29

**Authors:** Eduard Pavelić, David Glavaš Weinberger, Martin Čemerin, Eduard Rod, Dragan Primorac

**Affiliations:** 1 St. Catherine Specialty Hospital, Ulica Kneza Branimira 71E, Zagreb, Croatia; 2 Medical School, University of Split, Šoltanska Ulica 2, Split, Croatia; 3 Faculty of Medicine, Josip Juraj Strossmayer University of Osijek, Ulica Josipa Huttlera 4, Osijek, Croatia; 4 Faculty of Dental Medicine and Health, Josip Juraj Strossmayer University of Osijek, Crkvena Ulica 21, Osijek, Croatia; 5 Medical School, University of Rijeka, Ulica braće Branchetta 20/1, Rijeka, Croatia; 6 Medical School REGIOMED, Gustav-Hirschfeld-Ring 3, Coburg, Germany; 7 Eberly College of Science, The Pennsylvania State University, 517 Thomas Building, University Park, PA USA; 8 The Henry C. Lee College of Criminal Justice and Forensic Sciences, University of New Haven, 300 Boston Post Road, West Haven, CT USA; 9 Department of Orthopedics and Traumatology, St. Catherine Specialty Hospital, Ulica Kneza Branimira 71E, 10000 Zagreb, Croatia

**Keywords:** Case report, Septic, Reactive, Arthritis, *Clostridium difficile*, COVID-19, SARS-CoV-2

## Abstract

**Background:**

Reactive arthritis and septic arthritis rarely present concomitantly in the same joint and patient. Reactive arthritis presenting after coronavirus disease 2019 is also exceedingly rare, with less than 30 cases reported thus far. Less common pathogens such as *Clostridium difficile* have been reported to cause reactive arthritis, especially in patients with a positive human leukocyte antigen B27, and therefore should be considered in diagnostic algorithms. The aim of this case report is to highlight the difficulties and precautions in discerning and diagnosing patients presenting with sudden swelling of the knee.

**Case presentation:**

We report the case of a 70-year-old Caucasian male with a recent history of coronavirus disease 2019 upper respiratory infection and diarrhea and negating trauma, who presented with a swollen and painful knee. Pain and swelling worsened and inflammatory parameters increased after an intraarticular corticosteroid injection. The patient was therefore treated with arthroscopic lavage and intravenous antibiotics for suspected septic arthritis. Synovial fluid and synovium samples were taken and sent for microbiological analysis. Synovial fluid cytology showed increased leukocytes at 10,980 × 10^6^/L, while polymerase chain reaction and cultures came back sterile. *Clostridium difficile* toxin was later detected from a stool sample and the patient was treated with oral vancomycin. The patient was tested for the presence of human leukocyte antigen B27, which was positive. We present a review of the literature about the challenges of distinguishing septic from reactive arthritis, and about the mechanisms that predispose certain patients to this rheumatological disease.

**Conclusions:**

It is still a challenge to differentiate between septic and reactive arthritis of the knee, and it is even more challenging to identify the exact cause of reactive arthritis. This case report of a human leukocyte antigen-B27-positive patient highlights the necessity of contemplating different, less common causes of a swollen knee joint as a differential diagnosis of an apparent septic infection, especially in the coronavirus disease 2019 era. Treating the patient for septic arthritis prevented any possible complications of such a condition, while treating the *C. difficile* infection contributed to the substantial relief of symptoms.

## Background

Septic arthritis of the knee is a condition characterized by the presence of bacteria inside the knee joint that is accompanied by a severe inflammatory reaction. Epidemiological data have shown that the incidence of septic arthritis spans 2–10 per 100,000 patients [1]. Such a condition can be hazardous for the patient, with a loss of cartilage seen as early as 8 h after infection [2]. However, the correct diagnosis of septic arthritis can be a challenging task, as other conditions may present with similar clinical findings. The following is a case report of a 70-year-old Caucasian male who presented with recurrent knee pain symptoms after an undiagnosed severe acute respiratory syndrome coronavirus 2 (SARS-CoV-2) infection that necessitated arthroscopic lavage for high clinical and biological suspicion of septic arthritis.

## Case presentation

**Fig. 1:**
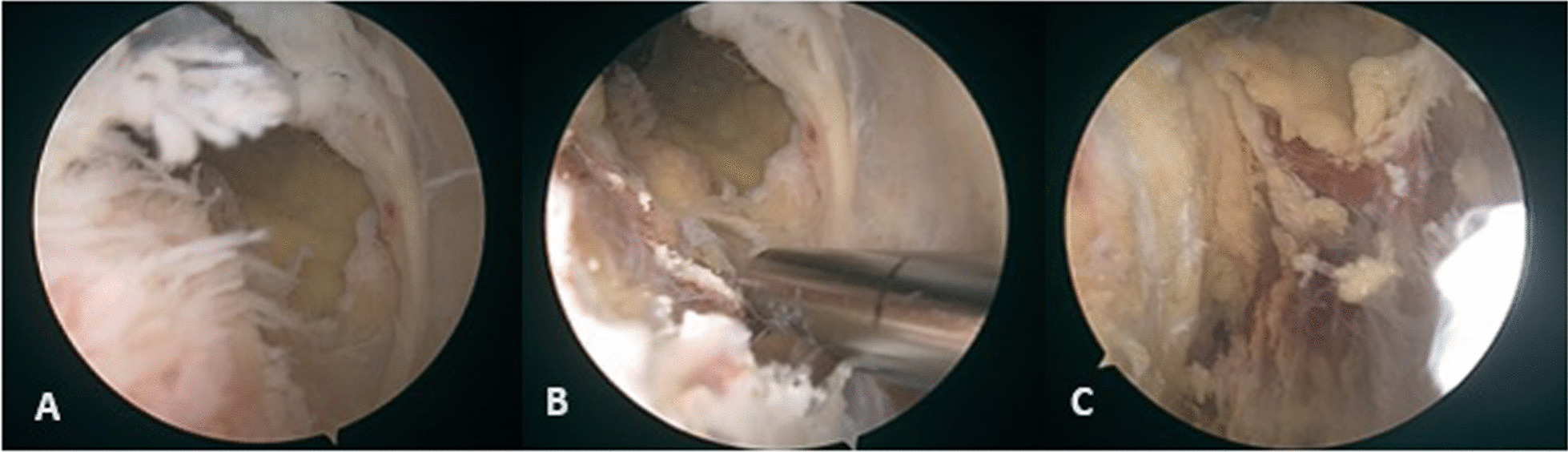
Arthroscopy images taken during emergent arthroscopic lavage of the patient’s knee. **a** Arthroscopy image shows slight synovitis at the injection site with petechial bleeding. **b** Arthroscopy image after debridement of the synovitis. **c** Arthroscopy image revealing bleeding and necrosis at the injection site

A 70-year-old Caucasian male patient with a history of partial meniscectomy of the left knee presented to the hospital on two occasions over a period of 10 days with complaints of swelling and pain in the knee while negating any trauma or other provoking factors. During the first visit, after a synovial fluid puncture, the patient received an intraarticular corticosteroid injection, which provided relief of symptoms for a few days. More intense knee swelling was present on the second visit 7 days after the first visit. Synovial fluid was collected from the knee and was sent for analysis. A follow-up appointment was performed 3 days after the second visit, and synovial fluid analysis results revealed increased leukocytes at 10,980 × 10^6^/L. Still, the microbiological analysis was negative for the presence of bacteria. Blood results showed increased leukocytes at 20 × 10^9^/L, C-reactive protein (CRP) at 222.7 mg/L, and procalcitonin at 0.33 μg/L. The patient was admitted to the hospital on the same day for suspicion of septic arthritis. Laboratory workup confirmed a previous coronavirus disease 2019 (COVID-19) infection via analysis of cellular immunity levels, which was not recognized a month prior to hospitalization and was treated with empiric antibiotics (amoxicillin and clavulanic acid) by an ear nose throat (ENT) specialist in a different institution prior to hospitalization. The patient underwent urgent arthroscopic lavage the day after admission, revealing a cloudy synovial fluid, moderate synovitis, petechial bleeding, some fibrin accumulations, and degenerative lesions of the medial meniscus and articular cartilage (Fig. 1). The synovial fluid sample taken on the day of admission and tissue samples taken during the arthroscopy were sent for bacterial culture analysis and were found to be sterile. Furthermore, panbacterial multiplex 16 s-polymerase chain reaction (PCR) was performed on the synovial fluid and tissue samples taken intraoperatively and showed negative results for the presence of bacterial DNA as the causative agent of septic arthritis (Table 1). On the first postoperative day, *Clostridium difficile* toxin was isolated from the stool and oral vancomycin was introduced in part with the empiric therapy for suspected septic arthritis. Follow-up laboratory parameters were carried out several times and showed regressive dynamics of inflammatory indicators over the next 2 days. The patient was discharged on the fourth day after admission, afebrile and not complaining of knee pain. Intravenous vancomycin (1 g twice a day) and ceftriaxone (2 g once a day) for potential septic arthritis with concomitant oral vancomycin (125 mg four times a day) for *C. difficile* were continued for 2 weeks postoperatively, followed by a switch to 1 week of oral cephalexin (500 mg three times a day). Furthermore, several laboratory parameters, such as serum levels of rheumatoid factor (RF) and anti-cyclic citrullinated peptide (anti-CCP) antibody levels, were evaluated, and human leukocyte antigen (HLA) testing was performed. Both anti-CCP antibodies (1.5 U/mL) and RF (< 20.0 kU/L) were below the reference interval values and therefore considered negative. The results of genetic testing for HLA revealed the presence of the HLA-B27 allele. At the follow-up visit 2 months after discharge, *C. difficile* toxin in the stool was tested and the result came back negative. The patient reported significant improvement in symptoms, negated any further episodes of diarrhea, and clinically showed signs of recovery.

**Table 1:** Panel of the pathogens tested using multiplex 16 s rDNA PCR

Gram-negative bacteria *Acinetobacter calcoaceticus*—*baumannii* complex, *Bacteroides fragilis*, *Enterobacterales*, *Enterobacter cloacae* complex, *Escherichia coli*, *Klebsiella aerogenes*, *Klebsiella oxytoca*, *Klebsiella pneumoniae* group, *Proteus* spp., *Salmonella*, *Serratia marcescens*, *Haemophilus influenzae*, *Neisseria meningitidis*, *Pseudomonas aeruginosa*, *Stenotrophomonas maltophilia*
Gram-positive bacteria *Enterococcus faecalis*, *Enterococcus faecium*, *Listeria monocytogenes*, *Staphylococcus* spp., *Staphylococcus aureus*, *Staphylococcus epidermidis*, *Staphylococcus lugdunensis*, *Streptococcus* spp., *Streptococcus agalactiae*, *Streptococcus pneumoniae*, *Streptococcus pyogenes*
Fungi *Candida albicans*, *Candida auris*, *Candida glabrata*, *Candida krusei*, *Candida parapsilosis*, *Candida tropicalis*, *Cryptococcus neoformans*/*gattii*
Additional pathogens *Campylobacter* (*jejuni*, *coli*, and *upsaliensis*), *Clostridium difficile* (*toxin* A/B), *Yersinia enterocolitica*, *Vibrio cholerae*, *V. parahaemolyticus*, *V. vulnificus*, *Shigella*

## Discussion and conclusion

Septic or infectious arthritis (SA) is an acute joint inflammation caused by microorganisms introduced into the joint either hematogenously or by direct inoculation. It is mainly a clinical diagnosis, and in the adult population, the main culpable microorganism is *Staphylococcus aureus* [3]. It is important to note that there is no consensus on how to base the diagnosis of SA in adults on laboratory values such as peripheral blood leukocytes, synovial fluid (SF), white blood cell (WBC) count, or even gram staining of the SF. All of the tests mentioned above are of moderate relevance when it comes to diagnosing the presence of a joint infection correctly [4, 5]. The role of PCR in the diagnostic algorithm is also conflictual and has to be backed by further studies [4, 6, 7].

This can make the diagnosis challenging even for highly skilled and experienced physicians. The distinction between septic and inflammatory noninfectious arthritis cannot be made on the basis of the mere observation of synovial fluid (SF). Even though literature reports that a SF WBC count of > 50.000/mm^3^ is highly indicative of infectious arthritis, this is also not a specific enough parameter for the diagnosis since numerous cases of SA have been reported with an inferior SF WBC count and gram staining that was negative for the presence of bacteria [8, 9]. The case fatality in patients with monoarticular disease, even in expert hands, is around 11%. Therefore, any patient with suspected SA should be admitted to the hospital for prompt assessment, diagnostics, and intravenous antibiotic treatment [3, 10].

Reactive arthritis (ReA), on the other contrary, is an acute non-purulent arthritis that classically presents as either sacroiliitis, enthesitis, asymmetrical oligoarthritis primarily of the lower limbs, or a combination of the above-mentioned features. It also has typical extraarticular manifestations such as urethritis, conjunctivitis, and mucocutaneous lesions of the genitalia (balanitis, keratoderma blenorrhagicum). ReA belongs to the spectrum of spondyloarthritides (SpA), which also includes ankylosing spondylitis (AS), psoriatic arthritis (PsA), and inflammatory bowel disease related arthritis (IBDa). ReA should be considered in the differential diagnosis of acute knee pain with effusion. It most commonly affects individuals 18–40 years of age. It is known to generally occur after genitourinary or gastrointestinal infections caused by microorganisms such as *Chlamydia trachomatis*, *Salmonella enteritidis, Shigella shigae, Yersinia enterocolitica*, and *Campylobacter jejuni* [11]. Individuals with the HLA-B27 allele or family history of ankylosing spondylitis have an increased risk of developing ReA [11]. Approximately 30–80% of ReA cases in population studies are reported in patients with a positive HLA-B27 antigen, which is also linked to other forms of the SpA spectrum such as AS and IBDa [12]. HLA-B27 is also linked with increased intestinal permeability, which may be a pathogenic mechanism for the above-mentioned pathogens [13, 14]. *Clostridium difficile* is a pathogen that is very rarely associated with ReA, and some viral pathogens such as parvovirus B19, chikungunya virus, hepatitis B, human immunodeficiency virus (HIV), and more recently, SARS-CoV-2 have been linked to post-viral ReA [13, 15–17]. Other viruses, including hepatitis C, rubella, mumps, Epstein–Barr virus (EBV), cytomegalovirus (CMV), herpes simplex virus (HSV) 1 and 2, and flavivirus, have been linked with arthritis as well, although the presentation of the disease mediated by these pathogens is generally acute and polyarticular [18]. It is believed that circulating antigens from the above-mentioned pathogens enter the synovium and subsequently cause the activation of synovial fluid mononuclear cells (SFMC) such as macrophages and CD4+ T-helper cells [19]. Circulating lipopolysaccharides from the bacterial membrane, microorganism DNA, and molecular mimicry of these bacterial antigens with the HLA-B27 epitope are believed to be a causing mechanism for ReA [20, 21]. Several authors describe other mechanisms for the pathogenesis of ReA for different bacterial microorganisms. *Chlamydia trachomatis* persists in an aberrant metabolically active form in peripheral monocytes that reach the synovium through the bloodstream from the primary site of infection [22]. In post-enteric ReA caused by invasive bacteria, there is no persistence of entire microorganisms, but rather, there is a presence of some of their structural components: outer membrane proteins (OMPs) such as porins, lipoproteins, or lipopolysaccharides of the outer membrane in the synovium and SF [23]. Both the aberrant form of *C. trachomatis* and the outer membrane antigens of enteric bacteria are subsequently presented to naive T-lymphocytes. This is thought to be the reason for increased SF and serum levels of cytokines such as IFN-γ, IL-1β, IL-4, IL-17, and TNF-α and the ensuing inflammation [12, 22]. The pathogenic mechanism for *C. difficile* ReA is still quite unclear, since the bacteria is enterotoxic (toxin A, toxin B) but not invasive. Toxin A contributes to the increased permeability of the intestinal wall through inactivation of host GTPases and therefore altering the mucosa. This finally allows for bacterial antigens to reach the bloodstream [13, 24]. This is a potential explanation as to how synovial fluid and knee swelling could occur post-*C. difficile* infection.

Since the breakout of COVID-19, less than 30 cases that fill the diagnostic criteria for post-viral ReA caused by SARS-CoV-2 have been reported, and the exact mechanism of this condition is still a topic for discussion [17, 25, 26]. Cellular immunity to COVID-19 is marked by a strong CD4+ TH1 cell response, secreting high levels of IFN-γ [27]. Some studies have proven that the synovial inflammation in ReA is primarily mediated by synovial CD4+ TH2 cells secreting interleukin-10, which consequently decreases the TH1 secretion of IFN-γ in the acute phase [12, 22, 28]. This correlates with a longer persistence of microorganisms in the SF [28–30]. No studies have yet been conducted on the effects of SARS-CoV-2 antigens on SFMC. It is possible that SARS-CoV-2 shares a myeloid cell activation pathway with other rheumatological diseases, which may contribute to some of its arthritic presentations, though rare, and to the elevated neutrophil count in the SF [31].

The patient developed arthritic symptoms 3.5 weeks after a COVID-19 upper respiratory tract infection that was treated with amoxicillin and clavulanic acid. The patient also reported post-antimicrobial diarrhea lasting for a week, which was highly probably caused by *C. difficile*. Increased CRP levels (222.7 mg/L), backed by an elevated procalcitonin, are regarded as highly indicative of an active bacterial infection and alerts to the possibility of a septic joint infection in cases of a painful and debilitating joint effusion. Relapsing joint effusions, over a period of 10 days after the intraarticular corticosteroid injections, indicate the possibility that the arthritis was not caused exclusively due to a reactive, rheumatologic condition. Treatment with intravenous antibiotics (vancomycin and ceftriaxone) for the plausible SA, and oral vancomycin for the eradication of *C. difficile*, were initiated immediately to cover both of the potential causes of arthritis. The antibiotic therapy was well tolerated by the patient; adverse effects included only slight nausea. Such intervention was necessary for the prevention of possible devastating effects of SA on the knee joint while allowing for an adequate diagnostic evaluation of other conditions included in the differential. As the HLA testing revealed the presence of HLA-B27 antigen, commonly associated with ReA, and since the results of both panbacterial PCR and bacterial culture of the synovial fluid and the synovium came back negative, ReA emerged as an even more plausible cause of the symptoms. However, the quick regression of knee pain and swelling, as well as the immediate drop in CRP levels after antibiotic therapy, does not allow us to exclude SA as a potential cause.

The distinction between diagnoses remains unclear, and so too the pathogen possibly causing ReA; whether it is SARS-CoV-2 or *C. difficile*. However, significant improvement in symptoms 2 months after discharge shows that the therapy was efficacious. Nevertheless, the lack of a histopathological analysis of a tissue biopsy prevents a definitive diagnosis of reactive arthritis. However, the absence of an isolated microorganism is most probably indicative of reactive arthritis. Synovial fluid results were neither in favor of septic nor reactive arthritis. As a result, regarding and treating the patient as a patient with SA prevented any complications of a possible SA infection. This case shines a light on the necessity to consider ReA in the differential diagnosis of joint effusion. It is also indispensable to contemplate the possibility of ReA presenting after infections with less common pathogens, such as *C. difficile* and SARS-CoV-2.

## Data Availability

The datasets used and/or analyzed during the current study are available from the corresponding author on reasonable request.

## Abbreviations

SA: Septic arthritis
SF: Synovial fluid
WBC: White blood cells
ReA: Reactive arthritis
SpA: Spondyloarthritis
AS: Ankylosing spondylitis
PsA: Psoriatic arthritis
IBDa: Inflammatory bowel disease related arthritis
SFMC: Synovial fluid mononuclear cells
OMPs: Outer membrane proteins

## References

[1] Vassallo C, Borg AA, Farrugia D, Mercieca C (2020). The epidemiology and outcomes of septic arthritis in the Maltese islands: a hospital-based retrospective cohort study. Mediterr J Rheumatol.

[2] Elsissy JG, Liu JN, Wilton PJ, Nwachuku I, Gowd AK, Amin NH (2020). Bacterial septic arthritis of the adult native knee joint. JBJS Rev.

[3] Mathews CJ, Weston VC, Jones A, Field M, Coakley G (2010). Bacterial septic arthritis in adults. Lancet.

[4] Carpenter CR, Schuur JD, Everett WW, Pines JM (2011). Evidence-based diagnostics: adult septic arthritis. Acad Emerg Med.

[5] Long B, Koyfman A, Gottlieb M (2019). Evaluation and management of septic arthritis and its mimics in the emergency department. West J Emerg Med.

[6] Yang S, Ramachandran P, Hardick A, Hsieh Y-H, Quianzon C, Kuroki M, Hardick J, Kecojevic A, Abeygunawardena A, Zenilman J (2008). Rapid PCR-based diagnosis of septic arthritis by early gram-type classification and pathogen identification. J Clin Microbiol.

[7] Jacquier H, Fihman V, Amarsy R, Vicaut E, Bousson V, Cambau E, Crémieux A-C, Delcey V, Hannouche D, Kaci R (2019). Benefits of polymerase chain reaction combined with culture for the diagnosis of bone and joint infections: a prospective test performance study. Open Forum Infect Dis.

[8] McGillicuddy DC, Shah KH, Friedberg RP, Nathanson LA, Edlow JA (2007). How sensitive is the synovial fluid white blood cell count in diagnosing septic arthritis?. Am J Emerg Med.

[9] El-Gabalawi HS, Firestein GS, Budd RC, Gabriel SE, McInnes IB, O'Dell JR (2013). Kelley’s textbook of rheumatology. Kelley’s textbook of rheumatology.

[10] Coakley G, Mathews C, Field M, Jones A, Kingsley G, Walker D, Phillips M, Bradish C, McLachlan A, Mohammed R (2006). BSR & BHPR, BOA, RCGP and BSAC guidelines for management of the hot swollen joint in adults. Rheumatology.

[11] Fauci, A.; Langford, C. *Harrison’s Rheumatology*; 4th ed.; McGraw Hill / Medical;, 2016;

[12] Lucchino B, Spinelli FR, Perricone C, Valesini G, Di Franco M (2019). Reactive arthritis: current treatment challenges and future perspectives. Clin Exp Rheumatol.

[13] Putterman C, Rubinow A (1993). Reactive arthritis associated with *Clostridium difficile* pseudomembranous colitis. Semin Arthritis Rheum.

[14] Kerr SW, Wolyniec WW, Filipovic Z, Nodop SG, Braza F, Winquist RJ, Noonan TC (1999). Repeated measurement of intestinal permeability as an assessment of colitis severity in HLA-B27 transgenic rats. J Pharmacol Exp Ther.

[15] Marwat A, Mehmood H, Hussain A, Khan M, Ullah A, Joshi M (2018). *Clostridium difficile* colitis leading to reactive arthritis: a rare complication associated with a common disease. J Investig Med High Impact Case Reports.

[16] Birnbaum J, Bartlett JG, Gelber AC (2008). *Clostridium difficile*: an under-recognized cause of reactive arthritis?. Clin Rheumatol.

[17] Slouma M, Abbes M, Mehmli T, Dhahri R, Metoui L, Gharsallah I, Louzir B (2022). Reactive arthritis occurring after COVID-19 infection: a narrative review. Infection.

[18] Tiwari V, Bergman MJ (2022). Viral arthritis.

[19] Carter JD, Hudson AP (2009). Reactive arthritis: clinical aspects and medical management. Rheum Dis Clin North Am.

[20] Alvarez-Navarro C, Cragnolini JJ, Dos Santos HG, Barnea E, Admon A, Morreale A, López JA (2013). Novel HLA-B27-restricted epitopes from *Chlamydia trachomatis* generated upon endogenous processing of bacterial proteins suggest a role of molecular mimicry in reactive arthritis. J Biol Chem.

[21] Cheeti A, Chakraborty RK, Ramphul K. Reactive arthritis; 2022. 29763006

[22] Zeidler H, Hudson AP (2014). New insights into chlamydia and arthritis. Promise of a cure?. Ann Rheum Dis.

[23] Singh R, Shasany AK, Aggarwal A, Sinha S, Sisodia BS, Khanuja SPS, Misra R (2007). Low molecular weight proteins of outer membrane of *Salmonella typhimurium* are immunogenic in Salmonella induced reactive arthritis revealed by proteomics. Clin Exp Immunol.

[24] Kasendra M, Barrile R, Leuzzi R, Soriani M (2014). *Clostridium difficile* toxins facilitate bacterial colonization by modulating the fence and gate function of colonic epithelium. J Infect Dis.

[25] Slouma M, Abbes M, Kharrat L, Gharsallah I (2022). Post-COVID-19 reactive arthritis. Clin Rheumatol.

[26] Shimoyama K, Teramoto A, Murahashi Y, Takahashi K, Watanabe K, Iba K, Yamashita T (2022). Surgically treated reactive arthritis of the ankle after COVID-19 infection: a case report. J Infect Chemother.

[27] Primorac D, Vrdoljak K, Brlek P, Pavelić E, Molnar V, Matišić V, Erceg Ivkošić I, Parčina M (2022). Adaptive immune responses and immunity to SARS-CoV-2. Front Immunol.

[28] Eliçabe RJ (2014). Immunopathogenesis of reactive arthritis: role of the cytokines. World J Immunol.

[29] Yin Z, Braun J, Neure L, Wu P, Liu L, Eggens U, Sieper J (1997). Crucial role of interleukin-10/interleukin-12 balance in the regulation of the type 2 T helper cytokine response in reactive arthritis. Arthritis Rheum.

[30] Thiel A, Wu P, Lauster R, Braun J, Radbruch A, Sieper J (2000). Analysis of the antigen-specific T cell response in reactive arthritis by flow cytometry. Arthritis Rheum.

[31] MacDonald L, Alivernini S, Tolusso B, Elmesmari A, Somma D, Perniola S, Paglionico A, Petricca L, Bosello SL, Carfì A (2021). COVID-19 and RA share an SPP1 myeloid pathway that drives PD-L1+ neutrophils and CD14+ monocytes. JCI Insight.

